# Hydrogel Membranes from Chitosan-Fish Gelatin-Glycerol for Biomedical Applications: Chondroitin Sulfate Incorporation Effect in Membrane Properties

**DOI:** 10.3390/gels9110844

**Published:** 2023-10-25

**Authors:** Andreas Karydis-Messinis, Dimitrios Moschovas, Maria Markou, Kyriaki Tsirka, Christina Gioti, Eleni Bagli, Carol Murphy, Aris E. Giannakas, Alkis Paipetis, Michael A. Karakassides, Apostolos Avgeropoulos, Constantinos E. Salmas, Nikolaos E. Zafeiropoulos

**Affiliations:** 1 Department of Material Science and Engineering, University of Ioannina, 45110 Ioannina, Greece; dmoschov@uoi.gr (D.M.); ktsirka@uoi.gr (K.T.); christina.a.gioti@gmail.com (C.G.); paipetis@uoi.gr (A.P.); mkarakas@uoi.gr (M.A.K.); aavger@uoi.gr (A.A.); 2 Biomedical Research Institute (BRI)-FORTH, 45110 Ioannina, Greece; mmarkou92@gmail.com (M.M.); elenibgl@hotmail.com (E.B.); carol_murphy@bri.forth.gr (C.M.); 3 Department of Food Science and Technology, University of Patras, 30100 Agrinio, Greece; agiannakas@upatras.gr

**Keywords:** chitosan, fish gelatin, chondroitin sulfate, hydrogel membranes, wound dressing, in vitro cell colonization, gelatin glycerol, biomedical applications, membranes, biomaterials

## Abstract

Chondroitin sulfate (ChS), chitosan (Chi), and fish gelatin (FG), which are byproducts of a fish-treatment small enterprise, were incorporated with glycerol (Gly) to obtain dense hydrogel membranes with reduced brittleness, candidates for dressing in wound healing applications. The mechanical properties of all samples were studied via Dynamic Mechanical Analysis (DMA) and tensile tests while their internal structure was characterized using Attenuated Total Reflectance-Fourier Transform Infrared Spectroscopy (ATR-FTIR) and X-ray Diffraction (XRD) instruments. Their surface morphology was analyzed by ThermoGravimetric Analysis (TGA) method, while their water permeability was estimated via Water Vapor Transmission Rate (WVTR) measurements. Wettability and degradation rate measurements were also carried out. Characterization results indicated that secondary interactions between the natural polymers and the plasticizer create the hydrogel membranes. The samples were amorphous due to the high concentration of plasticizer and the amorphous nature of the natural polymers. The integration of ChS led to decreased decomposition temperature in comparison with the glycerol-free sample, and all the materials had dense structures. Finally, the in vitro endothelial cell attachment studies indicate that the hydrogel membranes successfully support the attachment and survival of primary on the hydrogel membranes and could be appropriate for external application in wound healing applications as dressings.

## 1. Introduction

In recent years, wound management methods have evolved considerably due to the deeper understanding of the molecular and cellular processes that take place during the healing process and the factors controlling it. Consequently, the design and functionality of the wound healing patches has turned to the creation of multi-functional materials. Wounds are dissimilar in nature and characteristics and depend on a variety of factors, such as origin (burn, surgery, incision, etc.), the state of health of the person and, the manifestation of infection [1]. Therefore, the requirements of the wound for a wound dressing, depend entirely on its type. Modern patches must be biocompatible, non-cytotoxic, non-inflammatory, have a rate of degradation commensurate with the rate of new tissue formation and prevent/treat possible infections [2,3]. The most important property required for wound dressings and especially those intended for burns, is the ability to absorb and retain water. For this type of damage, it is necessary to keep the wound hydrated, to absorb its secretions and accelerate the healing process by avoiding cellular dehydration in order to promote collagen synthesis and angiogenesis [4]. Proper hydration increases the healing rate, protects the wound from infections, and reduces pain [5]. The synthetic process of the patches must be simple, fast, and economically advantageous. They must also provide mechanical stability to the wound and be easily sterilized [1].

Hydrogels are hydrophilic macromolecular networks, synthesized through the formation of physical and chemical crosslinking [6]. The characteristics of the hydrogels depend significantly on the polymers used (natural, synthetic or a combination of the two) and the interactions between them [7]. The formation of covalent bonds improves the mechanical properties; however, it leads to a reduction in the degradation rate, affecting the biocompatibility of the materials synthesized [8,9]. Physical crosslinking leads to the formation of a relatively weak network through molecular and/or secondary interactions such as electrostatic interactions, hydrogen bonds and hydrophobic interactions [7,10]. The disruption of the network in the case of physically crosslinked hydrogels can result from changes in various conditions (pH, temperature, solvent) giving these materials excellent properties (controlled release of bioactive agents and drugs). The main advantage of hydrogels synthesized through physical crosslinking, is biocompatibility because no crosslinker is required for the network formation and the potential toxicity problems that can be caused by cross-linkers (genipin, glutaraldehyde, etc.) used during chemical crosslinking are avoided [7,8]. Polysaccharide and protein-based materials have functional groups, such as hydroxyl groups, carboxyl groups and amino groups, providing binding sites appropriate to form secondary interactions. The weak interactions developed through physical crosslinking stabilize the network and the dissolution of the hydrogel is avoided.

Natural polymers like chitosan, gelatin and collagen are widely used in wound management due to their attractive properties (biodegradation, biocompatibility etc.) [11,12]. However, their use is limited to developing porous materials through complicated synthetic routes. The use of dense, non-porous hydrogel membranes in applications such as wound healing is also limited. Sharma et al. developed a polyelectrolyte complex (PEC) using chitosan and chondroitin sulfate for effective management of chronic wounds [13] Liu et al. developed biodegradable and cytocompatible coatings or the Prevention of Implant-Associated Infection [14]. Lu et al. developed chitosan hydrogels crosslinked with the synthetic polymer PEG as candidate antibacterial wound dressings [15].

In the present study hydrogel membranes were synthesized via a green process and characterized via various instrumental and in-vitro methods. These hydrogel membranes consisting of bio-based and bio-degradable materials such as Chitosan (Chi), Fish Gelatin (FG), Chondroitin Sulfate (ChS), and Glycerol (Gly). The novelty of this work is the development of dense hydrogel membranes by the incorporation of the ChS biomaterial in a ChS-free material developed in a previous published work [16], to further improve some properties. The comparison of these two groups of materials shown improvement of cell-adhesion and water-uptake properties, which are significant properties for wound healing applications, while the mechanical properties remain stable. The objective of the current work is to study the effect of ChS integration on the overall properties of the materials. ChS was selected because of its antioxidant, anti-inflammatory and anti-apoptotic properties [17,18]. ChS has a wide range of bioactivities including tissue regeneration, intracellular signaling, cell proliferation, and cell adhesion [19,20]. Fialkova et al. [21], reported that topical application of ChS on the wound surface of rats after surgery resulted in a significant reduction in edema of the tissue surrounding the wound compared to the wound where ChS was not used. It was also shown that the application of ChS led to a reduction in hyperemia and wound secretions in relation to wounds where ChS not applied. However, the use of ChS in wound healing applications is still limited.

## 2. Results and Discussion

### 2.1. ATR-FTIR Spectroscopy

**Figure 1:**
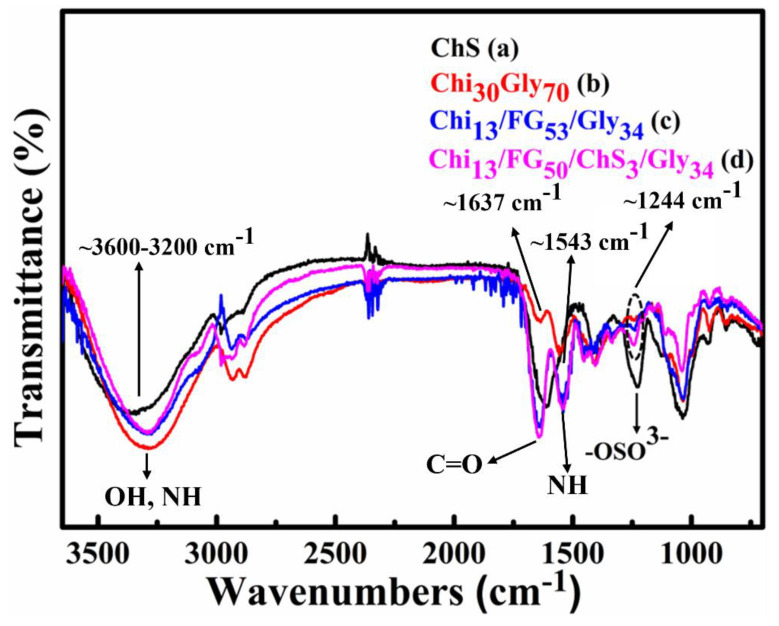
FTIR spectra of chondroitin sulfate (ChS) (**a**), chitosan/glycerol (Chi_30_Gly_70_) (**b**), chitosan/fish gelatin/glycerol membrane (Chi_13_FG_53_Gly_34_) (**c**) and chitosan/fish gelatin/chondroitin sulfate/glycerol membrane (Chi_13_FG_50_ChS_3_Gly_34_) (**d**).

Components’ inter- and intra-molecular secondary interactions were studied using an ATR-FTIR instrument. ChS, Chi_30_Gly_70_, Chi_13_FG_53_Gly_34_ (chitosan/fish-gelatin/glycerol), and Chi_13_FG_50_ChS_3_Gly_34_ spectra, obtaind from this instrument, are shown in Figure 1. Figure 1a presents the spectrum of the ChS membrane. The broad peak between 3600–3200 cm^−1^ arises from the stretching vibrations of the OH and NH groups [22]. The peak observed at 1610 cm^−1^ is representative of carbonyl groups (C=O). The peak at 1228 cm^−1^ is ascribed to then negatively charged group -OSO^3−^ of ChS [20]. The peaks at 1631 cm^−1^, 1637 cm^−1^ and 1637 cm^−1^ appear in Figure 1b, Figure 1c and Figure 1d respectively, attributed to stretching vibrations of C=O. The peaks revealed at 1553 cm^−1^, 1543 cm^−1^ and 1543 cm^−1^, ascribed to bending vibrations of N-H, while the peaks at 1240 cm^−1^, 1238 cm^−1^ and 1244 cm^−1^ indicate bending vibrations of N-H groups [23]. The observed shifts of the peaks in the spectra Figure 1c relative to the spectra Figure 1b, indicate hydrogen bonds formation between chitosan and fish gelatin [23,24,25]. The shifts of Figure 1d relative to the spectra Figure 1b, designate CONH_2_ formation (interactions between chitosan and chondroitin sulfate). The increased intensity of the peak at 1244 cm^−1^ in Figure 1d confirm the interactions between chondroitin sulfate and chitosan [20,23]. The observed peaks at 2926 cm^−1^ (Figure 1b), at 2936 cm^−1^ (Figure 1c) and 2934 cm^−1^ (Figure 1d) are representative of asymmetrical stretching vibrations of C-H groups while the peaks at 2879 cm^−1^ (Figure 1b), at 2876 cm^−1^ (Figure 1c) and 2883 cm^−1^ (Figure 1d) arises from symmetrical stretching vibrations of C-H groups of chitosan. The broad peaks between 3500–3000 cm^−1^ indicate the existence of stretching vibrations of O-H and N-H groups [23]. The shifts designate ionic interactions between the natural polymers and hydrogel membrane formation from polyelectrolytes.

### 2.2. XRD Analysis

**Figure 2:**
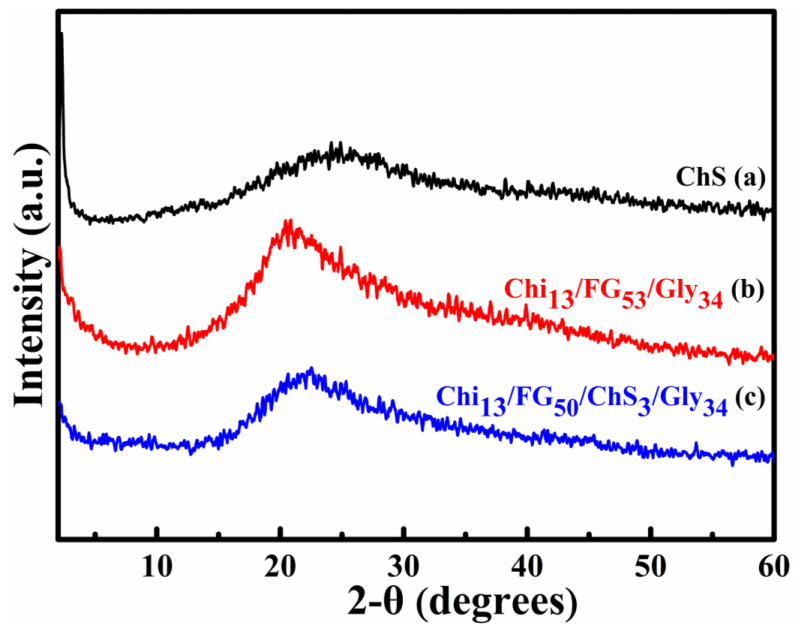
XRD diffractograms of ChS (**a**), Chi_13_FG_53_Gly_34_ (**b**) and Chi_13_FG_50_ChS_3_Gly_34_ (**c**).

The XRD patterns of ChS, Chi_13_FG_53_Gly_34_ and Chi_13_FG_50_ChS_3_Gly_34_ are shown in Figure 2. Figure 2a represents the XRD pattern of ChS. The broad peak appears at 2θ = 24°, confirms the amorphous nature of polysaccharide. Τhe amorphous nature of chondroitin sulfate ascribed to the low crystallinity profile of the main chain of the natural polymer [26,27,28]. Τhe rest of the materials showed a similar behavior (Figure 2b,c).The amorphous nature of the natural polymers utilized in the present work as well as the high concentration of the plasticizer, led to the development of amorphous materials [29].

### 2.3. TGA Measurements

**Figure 3:**
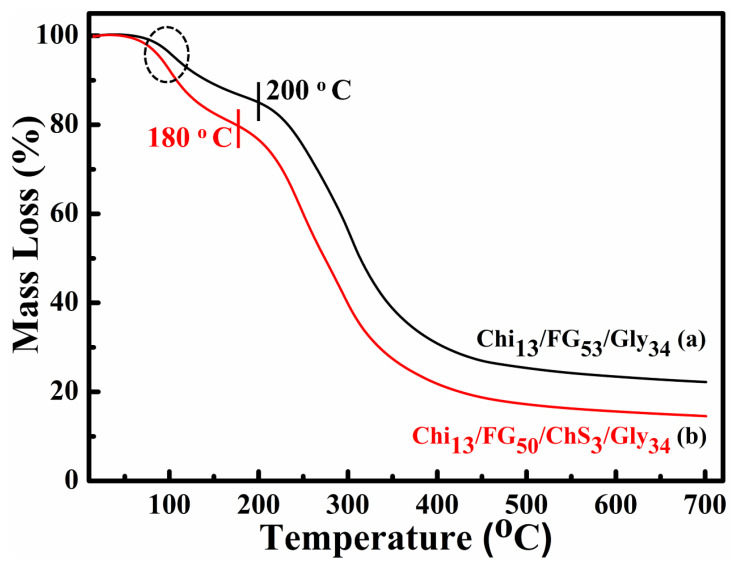
TGA thermograms of Chi_13_FG_53_Gly_34_ (**a**) and Chi_13_FG_50_ChS_3_Gly_34_ (**b**).

The results of the TGA measurements of Chi_13_FG_53_Gly_34_ and Chi_13_FG_50_ChS_3_Gly_34_ (before and after the integration of ChS are depicted in Figure 3. In all the thermograms a mass loss in the range between ~80–150 °C is observed, showing the materials’ water removal. A notable mass loss occurs in the temperature range 220–400 °C. This loss is ascribed to materials’ functional groups disintegration and because of this the materials’ disruption. Figure 3a shows the thermal decomposition of ChS-free material while Figure 3b the results of ChS-containing material, respectively. In the first case the decomposition begins at 200 °C while after the integration of chondroitin sulfate, begins at 180 °C. The addition of chondroitin sulfate led to decreased thermal stability perhaps due to poor miscibility of the components. The remaining 20–30% of the mass is ascribed to remaining ash.

### 2.4. DMA Measurements

**Figure 4:**
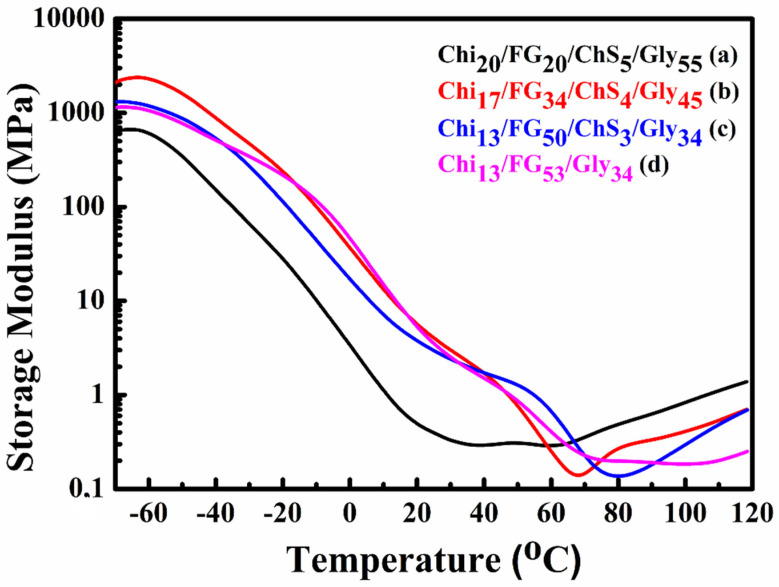
Chi_20_FG_20_ChS_5_Gly_55_ storage modulus and DMA results presented on graphs (**a**), Chi_17_FG_34_ChS_4_Gly_45_ (**b**), Chi_13_FG_50_ChS_3_Gly_34_ (**c**) and Chi_13_FG_53_Gly_34_ (**d**) hydrogel membrane.

Dynamic mechanical analysis was used to examine the thermomechanical response and the miscibility of the blends. In Figure 4 the results of the storage modulus as a function of temperature of the hydrogel membranes Chi_20_FG_20_ChS_5_Gly_55_ (Figure 4a), Chi_17_FG_34_ChS_4_Gly_45_ (Figure 4b), Chi_13_FG_50_ChS_3_Gly_34_ (Figure 4c) and Chi_13_FG_53_Gly_34_ (Figure 4d) are shown. All the materials showed a similar behavior in the range between −70–120 °C. The integration of chondroitin sulfate did not improve the mechanical properties of the materials, probably due to the low concentration used. Τhe observed increase in storage modulus after ~80 °C, is attributed to the removal of water which acts as a plasticizer in these materials [30].

**Figure 5:**
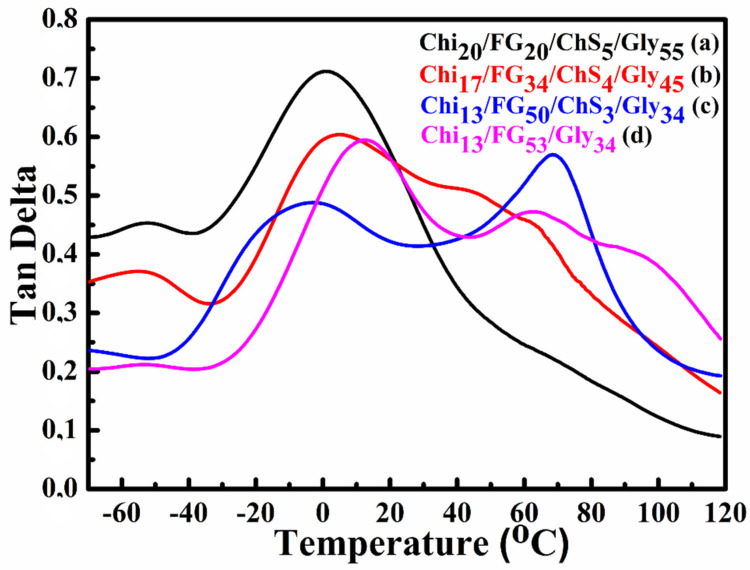
Tan delta plots of Chi_20_FG_20_ChS_5_Gly_55_ (**a**), Chi_17_FG_34_ChS_4_Gly_45_ (**b**), Chi_13_FG_50_ChS_3_Gly_34_ (**c**) and Chi_13_FG_53_Gly_34_ (**d**) hydrogel membrane.

Figure 5 shows the tan delta variation depending on temperature increase. The glass transition temperatures (T_g_s) of the hydrogel membranes Chi_20_FG_20_ChS_5_Gly_55_ (Figure 5a), Chi_17_FG_34_ChS_4_Gly_45_ (Figure 5b), Chi_13_FG_50_ChS_3_Gly_34_ (Figure 5c) and Chi_13_FG_53_Gly_34_ (Figure 5d) are located at 0.9 °C, 4.4 °C and −8.7 °C respectively. The peaks appear in the range between 40–80 °C at Figure 5b–d, are attributed to the poor miscibility of the natural polymers with the plasticizer and are the T_g_s of chitosan and fish gelatin which did not interact with chondroitin sulfate and glycerol. In Figure 5a only one T_g_ is observed, indicating the development of a single-phase system.

### 2.5. Tensile Properties

**Figure 6:**
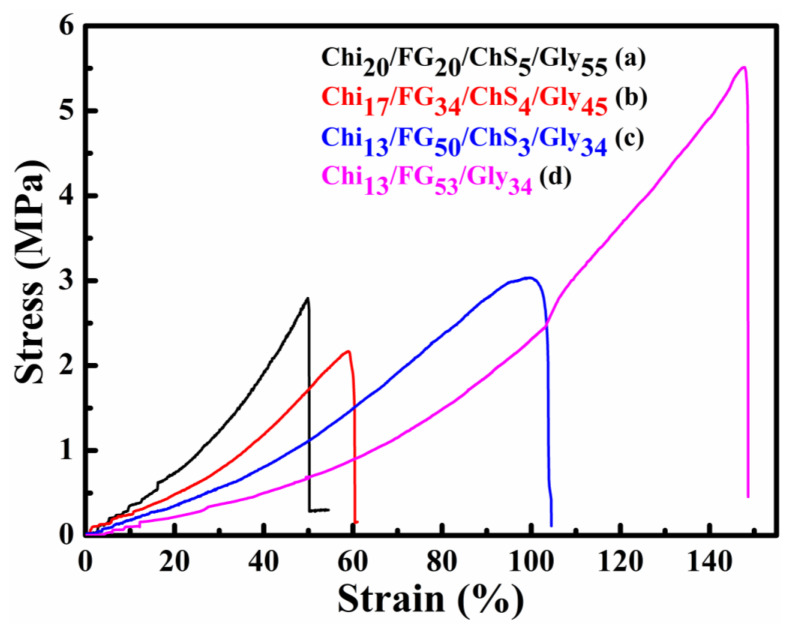
Indicative stress vs strain plots of the hydrogel membranes Chi_20_FG_20_ChS_5_Gly_55_ (**a**), Chi_17_FG_34_ChS_4_Gly_45_ (**b**), Chi_13_FG_50_ChS_3_Gly_34_ (**c**) and Chi_13_FG_53_Gly_34_ (**d**).

The average tensile strength and the respective strain to failure of the hydrogel membranes are summarized in Table 1 and indicative stress-strain plots from the tested membranes are presented in Figure 6. The Chi_13_FG_53_Gly_34_ can be regarded as the reference membrane with an average stress of 6.03 MPa and a strain to failure equal to 152.95%. According to the tensile tests the incorporation of ChS in the blend caused a decrease of both the strength and the strain to failure values of the membranes. The smallest deterioration in terms of strength was observed for the Chi_17_FG_34_ChS_4_Gly_45_ specimens in comparison to the reference membrane. On the other hand, the smallest decrease in the strain to failure values was observed for the Chi_13_FG_50_ChS_3_Gly_34_ in comparison to the reference membranes. An interesting observation is that although all the other membranes failed in an abrupt manner, as indicated by curves Figure 6a,b,d, whereby a maximum stress was observed and the specimens then ruptured suddenly, the Chi_13_FG_50_ChS_3_Gly_34_ specimens failed more gradually, sustaining the maximum stresses for an amount of strain. This different and more ductile failure type was consistently observed in all the tested specimens of this category. Literature values report normal human skin tensile strength in the range 2.5–16 MPa and strain at break percentage 70% [31]. Such results are in agreement with the experimental data of this study.

### 2.6. SEM Measurements

**Figure 7:**
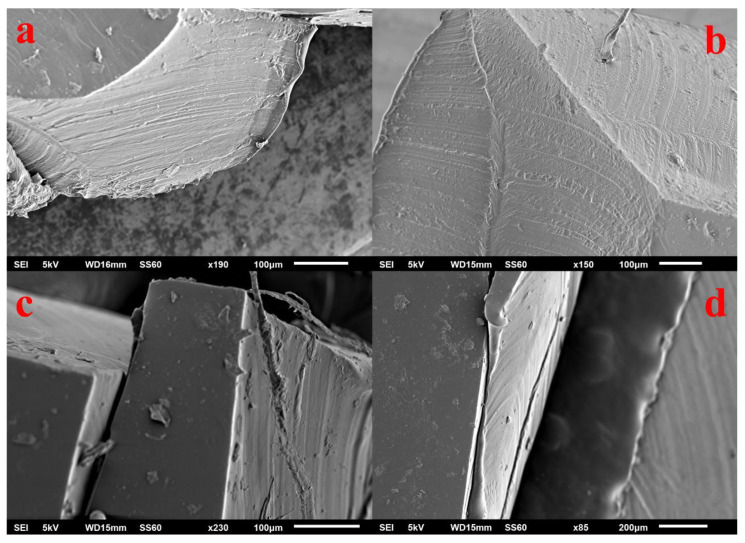
Cross sectional SEM images of Chi_20_FG_20_ChS_5_Gly_55_ (**a**), Chi_17_FG_34_ChS_4_Gly_45_ (**b**), Chi_13_FG_50_ChS_3_Gly_34_ (**c**) and Chi_13_FG_53_Gly_34_ (**d**) hydrogel membrane.

SEM was used to study the influence of the synthetic procedure and the incorporation of ChS on the surface morphology of the hydrogel membranes. Representative surface and cross section SEM images of the hydrogel membranes Chi_20_FG_20_ChS_5_Gly_55_ (a), Chi_17_FG_34_ChS_4_Gly_45_ (b), Chi_13_FG_50_ChS_3_Gly_34_ (c) and Chi_13_FG_53_Gly_34_ (d) are illustrated in Figure 7. All the samples show dense morphology, while no pores or voids are observed. The solvent evaporation method used to develop the hydrogel membranes is an effective method for the development of hydrogel membranes with continuous and dense structure.

### 2.7. Water Uptake

**Figure 8:**
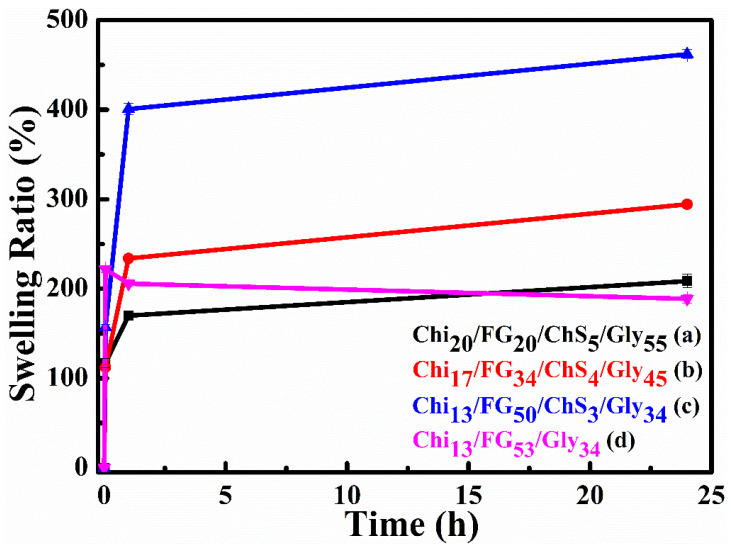
Swelling rate of Chi_20_FG_20_ChS_5_Gly_55_ (**a**), Chi_17_FG_34_ChS_4_Gly_45_ (**b**), Chi_13_FG_50_ChS_3_Gly_34_ (**c**) and Chi_13_FG_53_Gly_34_ (**d**) hydrogel membrane.

Water uptake constitutes an important characteristic in wound dressings because a moist environment is required to enhance wound healing and to avoid dehydration in the wound area [32]. In the present study, the water uptake results are expressed as Swelling Ratio (%) and are shown in Figure 8. The results verify the important role of chondroitin sulfate in water uptake ability. The swelling ratio of the hydrogel membranes Chi_20_FG_20_ChS_5_Gly_55_ (Figure 8a), Chi_17_FG_34_ChS_4_Gly_45_ (Figure 8b), Chi_13_FG_50_ChS_3_Gly_34_ (Figure 8c) and Chi_13_FG_53_Gly_34_ (Figure 8d) is 209%, 294%, 462% and 186%, respectively. Comparing the results depicted in Figure 8c with the results in Figure 8d, it is clear that the incorporation of chondroitin sulfate led to increased water uptake ability due to the hydrophilic nature of the natural polymer ChS. As can be seen from Table 2, the sample Chi_13_FG_50_ChS_3_Gly_34_ contains the highest concentration of fish gelatin. The higher water uptake capacity of that sample is attributed to the hydrophilicity of fish gelatin [16].

### 2.8. Degradation Rate

**Figure 9:**
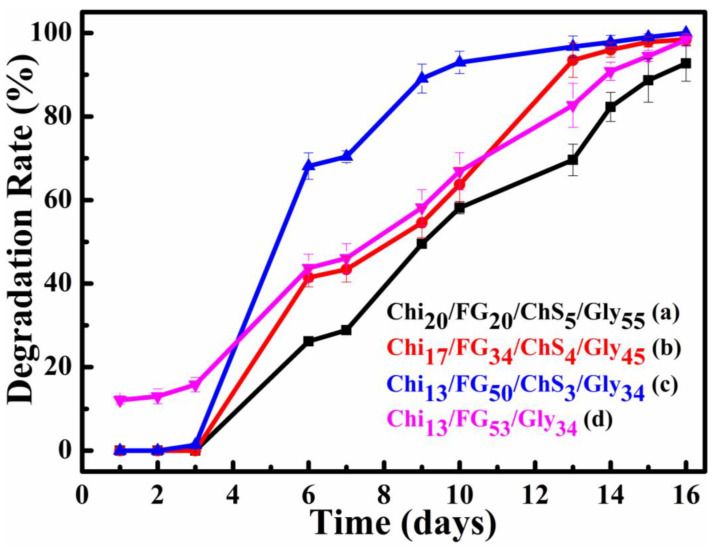
Degradation rate of Chi_20_FG_20_ChS_5_Gly_55_ (**a**), Chi_17_FG_34_ChS_4_Gly_45_ (**b**), Chi_13_FG_50_ChS_3_Gly_34_ (**c**) and Chi_13_FG_53_Gly_34_ (**d**) hydrogel membrane.

Degradation rate (%) results of the current study are presented in Figure 9. It is obvious from this figure that the higher the fish gelatin concentration the higher the degradation rates. This phenomenon occurs because of fish gelatin high solubility in water. The fish-gelatin concentration increase cause an increase of non-active functional groups in the blend. Thus, no hydrogen bonds formed with chitosan, chondroitin sulfate and glycerol groups. The degradation rates were further increased after the addition of ChS. The higher degradation rate (93% mass loss the 10th day) exhibited by the Chi_13_FG_50_ChS_3_Gly_34_ membrane while the ChS free membrane Chi_13_FG_53_Gly_34_ showed reduced degradation rate (67% mass loss the 10th day). The higher degradation rate observed for the Chi_13_FG_50_ChS_3_Gly_34_ relative to Chi_13_FG_53_Gly_34_ membrane is maybe attributed to poor miscibility of the blend components after the integration of ChS due to lack of binding sites.

### 2.9. Water Vapor Transmision Rate (WVTR)

**Figure 10:**
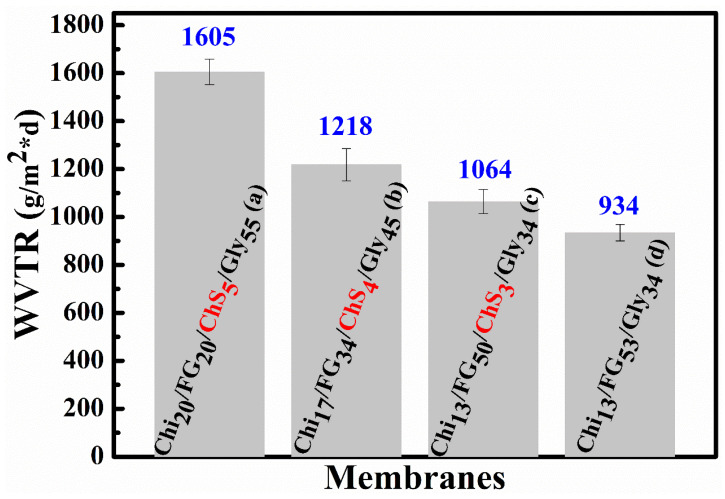
Water vapor transmission rate of Chi_20_FG_20_ChS_5_Gly_55_ (**a**), Chi_17_FG_34_ChS_4_Gly_45_ (**b**), Chi_13_FG_50_ChS_3_Gly_34_ (**c**) and Chi_13_FG_53_Gly_34_ (**d**) hydrogel membranes.

The results of Water Vapor Transmission Rate (WVTR) measurements interpretation are presented in Figure 10. For the Chi_20_FG_20_ChS_5_Gly_55_, Chi_17_FG_34_ChS_4_Gly_45_, Chi_13_FG_50_ChS_3_Gly_34_, and Chi_13_FG_53_Gly_34_ hydrogel membranes the WVTR values are 1605, 1218, 1064, and 934 g·m^−2^·d^−1^, respectively. The higher the fish gelatin concentration in the blends the lower the water vapor permeability. The incorporation of chondroitin sulfate with the fish gelatin increases the WVTR values comparing with the relevant WVTR values of the ChS free material (Figure 10c versus Figure 10d). The vapors permeate the ChiFGChSGly membranes through an adsorption and diffusion process [31]. According to the literature reports the WVTR values of human skin varies from 204 g·m^−2^·day^−1^ to 5138 ± 202 g·m^−2^·day^−1^ depending on the type and healing stage of the wound [1,31]. The WVTR values of the examined Chi_x_FG_x_ChS_x_SGly_x_ membranes were in the range of 1064 to 1605 g·m^−2^·day^−1^. Such values indicate the appropriation of the examined hydrogel membranes for wound dressing.

### 2.10. Attachment of Endothelial Cells on Hydrogel Membranes In Vitro

**Figure 11:**
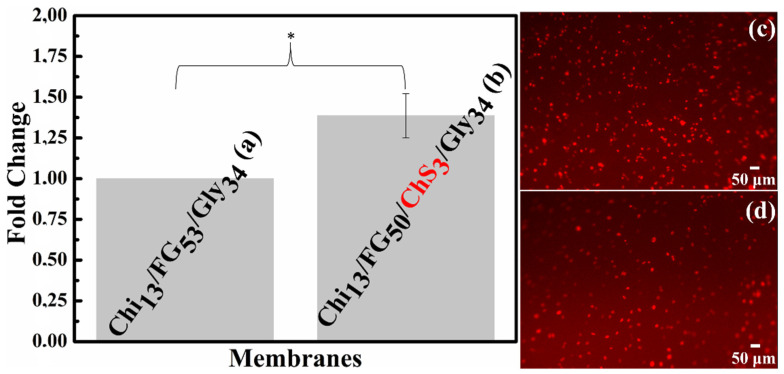
Attachment of Endothelial cells on Chi_13_FG_53_Gly_34_ (**a**) and Chi_13_FG_50_ChS_3_Gly_34_ (**b**) hydrogel membranes. Numbers of attached ECs at 4 h are expressed as fold change relative to Chi_13_FG_53_Gly_34_ membrane and presented as mean ± SD from three independent experiments. * *p* < 0.05. Images of ECs infected with H2B-mCherry lentivirus after 4h on hydrogel membranes Chi_13_FG_50_ChS_3_Gly_34_ (**c**) and Chi_13_FG_53_Gly_34_ (**d**).

To test the biocompatibility of the Chi_13_FG_53_Gly_34_ and Chi_13_FG_50_ChS_3_Gly_34_ hydrogel membranes, we addressed the attachment of primary endothelial cells. The choice of primary endothelial cells was dictated by the fact that (1) these cells are very sensitive and do not easily attach to all surfaces and (2) due to their application in regenerative medicine and tissue repair. Indeed, angiogenesis (the formation of blood vessels from pre-existing ones) is critical for tissue regeneration and normal tissue function, as blood vessels transport nutrients, oxygen and blood cells to all tissues, while removing waste materials and carbon dioxide. The endothelial cells form a single cell layer that lines all blood vessels and regulates exchanges between the bloodstream and the surrounding tissues, all cells are located within 100 to 200 μm of blood vessels—the diffusion limit for oxygen [33]. We found that endothelial cells attached successfully οn both hydrogel membranes 4h after their addition, providing strong evidence that there is no cytotoxicity and allowing the further use of the membranes in in vitro and in vivo experiments. EC. attachment to the Chi_13_FG_50_ChS_3_Gly_34_ membrane was statistically significantly higher (*p* < 0.05) compared to the Chi_15_FG_50_Gly_35_ membrane as shown in Figure 11. This finding is supported by the work of Thalla et al. reporting the incorporation of chondroitin sulfate improves membrane properties by increasing endothelial cell adhesion [34,35,36].

## 3. Conclusions

In the present study, hydrogel membranes of dense chitosan/fish gelatin/chondroitin sulfate/glycerol were synthesized. The solution casting-evaporation procedure was adopted for this synthesis and the use of chemical crosslinkers was rejected because of their potential cytotoxicity effects. Materials’ properties changed because of the chondroitin sulfate addition and the effect of its integration in the final product was evaluated. The increase of fish gelatin content as well as the incorporation of chondroitin sulfate in the blend, led to increased water uptake ability. Furthermore, the integration of chondroitin sulfate, led to increase in WVTR and degradation rate. The attachment of endothelial cells was supported by both the hydrogel membranes Chi_13_FG_53_Gly_34_ and Chi_13_FG_50_ChS_3_Gly_34_, confirming that there is no cytotoxicity, and the ChS containing membrane showed enhanced endothelial cell attachment. Finally, as indicated from the overall materials’ properties, the effective wound dressing and wound healing could be achieve using the product proposed in this study.

## 4. Materials and Methods

### 4.1. Materials

Sigma–Aldrich (St. Louis, MO, USA) was the supplier of medium molecular weight chitosan (75–85% deacetylated), cold water fish skin derived gelatin, glycerol, heparin, and dPBS (dulbecco’s Phosphate Buffered Saline). Acros Organics chondroitin sulfate sodium salt (Geel, Belgium) was also purchased for this project needs. Honeywell Fluka Research Chemicals (Charlotte, NC, USA) was the supplier for acetic acid while M199, fetal bovine serum (FBS) and penicillin-streptomycin were purchased from Gibco™—Thermo Fisher Scientific (Waltham, MA, USA). Calcein provided by eBioscience (San Diego, CA, USA) and lentivirus production H2B-mCherry plasmid provided from Addgene (Watertown, MA, USA) were also used for project’s purproses.

### 4.2. Hydrogel Membrane Synthesis

**Figure 12:**
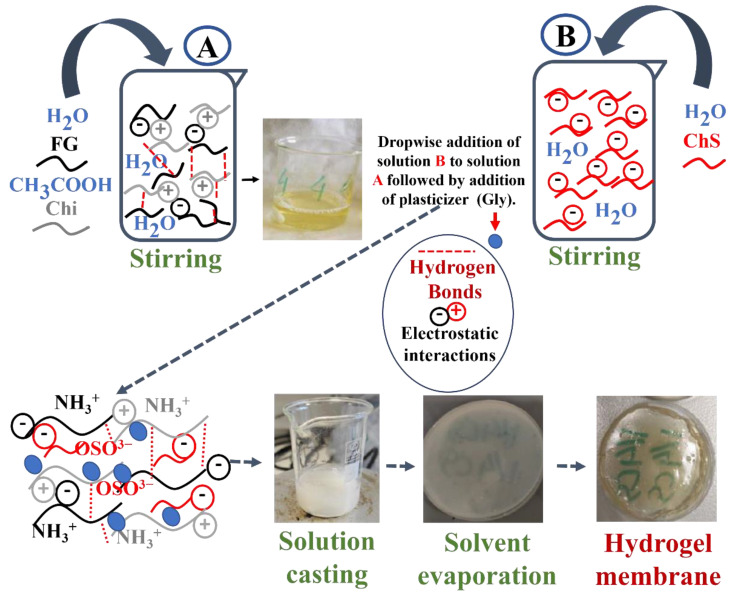
Schematic illustration of hydrogel membranes synthesis.

For the Chi_20_FG_20_ChS_5_Gly_55_ hydrogel membrane synthesis, in a beaker A (Figure 12) containing 19 mL of distilled water, 0.5 g of fish gelatin was added and allowed to stir until completely dissolved. Then, while stirring the solution, 0.5 g of chitosan was added followed by the addition of 4% (*v*/*v*) acetic acid. The solution was left to stir for 10 min. In a second beaker B (Figure 12), 0.125 g of chondroitin sulfate were dissolved in 5 mL of water and this solution was then added dropwise to beaker A (Figure 12), under stirring. After the addition of chondroitin sulfate the solution turned from pale yellow and clear to white translucent. The transformation of the solution from clear to translucent is attributed to the formation of polyelectrolyte [20]. Subsequently 4% (*v*/*v*) glycerol was added, and the solution remained under stirring overnight. The final solution contains 0.5 g (2% *w*/*v*) fish gelatin, 0.5 g (2% *w*/*v*) chitosan, 0.125 g (0.031% *w*/*v*) chondroitin sulfate and 1.26 g (4% *v*/*v*) glycerol. The solution is then placed in an ultrasonic bath for 30 min in degas mode to remove the bubbles. It is then transferred to polystyrene plates to evaporate the solvent and form the hydrogel membrane. The same procedure was followed for the synthesis of the rest of materials and the synthetic procedure of Chi_13_FG_53_Gly_34_ is the same but without the addition of ChS. The quantities used are listed in Table 2 and the synthesis is illustrated in Figure 12.

### 4.3. ATR-FTIR Analysis

A coupled instrument, of Jasco FT/IR-4100 spectrometer (JASCO, Interlab, S.A., Athens, Greece) and Jasco IRT-5000 microscope (JASCO, Interlab, S.A., Athens, Greece), was used to carry out ATR-FTIR measurements on the prepared membranes characterization. ATR device of the instrument includes a ZnSe prism with a 250 μm contact area, around 2.0 µm (@1000 cm^−1^) penetration depth, and capable for measurements down to 650 cm^−1^.

### 4.4. XRD Analysis

A PANalytical X’PertPRO diffractometer was employed for XRD crystallinity measurements using CoKα radiation. The X’Celerator detector of this instrument was operated at 40 kV voltage and 40 mA current. The 2θ range 2°–60° was scanned on all membrane samples.

### 4.5. Thermogravimetric Analysis (TGA)

Setsys Evolution- Setaram thermogravimetric instrument was used for TGA, TG-DSC, and TG-DTA analysis of ~30 mg of sample placed in a platinum crucible. All tested samples were heated from ambient to 700 °C with a temperature increasing rate of 10 °C·min^−1^ while the gas (N_2_) flow rate was set at 25 mL·min^−1^.

### 4.6. Dynamic Mechanical Analysis (DMA)

Thermomechanical properties were determined using a Q800 (TA Instruments, New Castle, DE, USA) instrument in film tension mode and a deformation amplitude set at 15 μm. Storage modulus (E′) and loss factor (tanδ) were estimated scanning the temperature range from −70 °C to 120 °C with a temperature increasing rate of 3 °C/min and a frequency of 1 Hz.

### 4.7. Mechanical Properties

ASTM D638 stadard was adopted to evaluate the tensile properties of the different membranes. A hand made horizontal tensile testing stage was used. Samples were cut to type V dumbbell shape and a 0.1 min^−1^ strain rate was applied to tested samples until failure. A linear variable differential transformer (LVDT) was used to record the elongation of each speciment. Three independent samples per each type of membrane were tested using a 44.5 N load cell (or a 445 N load cell for the pure specimens) to measure the load. According to theory and Equation (1), strain was calculated by dividing the elongation values with the initial effective length of each specimen.

(1) $$\mathrm{strain}=\frac{\Delta l}{l_{0}}$$

where Δ*ℓ* is the elongation value and *ℓ* the initial effective length.

Similarly, according to theory and Equation (2), stress was estimated by dividing the load values with the cross-sectional area of each specimen.

(2) $$\sigma =\frac{F}{S}$$

where F is the load applied to the sample for elongation and S is the cross-sectional area of the sample.

### 4.8. Scanning Electron Microscopy (SEM)

JEOL JSM-6510 LV (JEOL Ltd., Tokyo, Japan) SEM instrument was employed to characterize the surface structure of the samples by the applications of 5 kV acceleration voltage. For such characterizations an equiped X-Act EDS-detector (Abingdon, Oxford Instrunments, Oxfordshire, UK) was used. To avoid samples charging the membranes were firstly sputter-coated with gold in vacuum for 30 s, and a 5 kV acceleration voltage was applied for the examination tests.

### 4.9. Water Uptake Study

Circular disk shape samples of 12 mm diameter were tested for the water uptake ability. Membranes were immersed in distilled water and the swelling property was estimated by immediately weighing at 3 min, 1 h, and 24 h after the excess water removal using a filter paper as absorbant material. Equation (3) was used for the membranes’ swelling ability estimation.

(3) $$SR=\frac{W_{s}-W_{d}}{W_{d}}$$

where *SR* is the swelling ratio, *W_s_* is the weight of the membrane measured at each specific time point, and *W_d_* is the initial weight of the membrane. Swelling ratio (*SR*) is the indicative value of the swelling ability.

### 4.10. Degradation Rate Study

Degradation rate study of hydrogel membranes was carried out by systematic measuring of the weight loss of samples. Circular disk shape samples of 12 mm diameter and initial weight m_0_ were immersed in 5 mL of distilled water and weighted after the 1st, 2nd, 3rd, 6th, 7th, 9th, 10th, 13th, 14th, 15th and 16th day. Weighing carried out after the excess water removal with filter paper as absorbant. The (%) degradation rate was determined according to Equation (4).

(4) $$\%\;\mathrm{degradation}=\frac{m_{0}-m_{i}}{m_{0}}\times 100$$

where m_0_ is initial mass of the hydrogel membrane and m_i_ is the mass of the membrane at the end of the ith day.

### 4.11. Water Vapor Permeability

Membranes were placed on top of the circular mouth of glass bottles contained 5 mL of distilled water. The mouth perimeter was sealed using commercial glue and the water-vapor permeability of membranes was estimated according to a procedure reported in literature [31]. The water vapor transmission rate measurements were carried out at 37 °C and calculated according to the Equation (5).

(5) $$\mathrm{WVTR}=\frac{W_{i}-W_{0}}{t\cdot A}$$

where WVTR is the Water Vapor Transmission Rate (g·m^−2^·d^−1^), W_i_ is the weight of the handmade device at specific time t_i_, W_0_ is the initial mass of the system (t_0_ = 0), and A is the cross-sectional area of the glass bottle mouth.

### 4.12. Attachment of Endothelial Cells, In Vitro, on Hydrogel Membranes ECs Adhesion Assay

Human endothelial cells (ECs) originated from an umbilical vein (HUVECs) source via an isolation method which was described previously [33]. Such cells were cultivated in M199 basal medium using 20% fetal bovine serum and 30 μg/mL endothelial cell growth (ECGS) as supplement, 4.7 U/mL heparin and 1% penicillin-streptomycin. Cells were passaged at a ratio of 1 to 3 and were used up to passage 3. After the isolation cells were infected with a H2B-mCherry lentivirus, which imparts a red fluorescent nuclear signal. Lentiviral particles were prepared according to standard protocols. The Chi_13_FG_50_ChS_3_Gly_34_ and Chi_13_FG_53_Gly_34_ hydrogel membranes were cut into 6mm diameter pieces, washed twice for 30 min with dPBS (dulbecco’s Phosphate Buffered Saline and placed into a 24-well culture plate. 30,000 HUVECs infected with H2B-mCherry lentivirus were added to each well and cultured at 37 °C, 5% CO_2_. 4 h later the medium was changed to remove unattached cells and images of attached ECs were taken using a Leica DM IRBE fluorescence microscope. The number of attached cells was measured using ImageJ software. Three independent experiments were performed. The relative number of attached cells on the two polymeric hydrogel membranes from three independent experiments were expressed as mean ± the standard deviation value.

Statistical analysis was performed using SPSS 22.0 (SPSS, Inc). Normality tests were applied to all measurement variables. The one-way analysis of variance (ANOVA) was used for comparison between the two membranes and obtained significant probability values were also corrected for multiple testing (Bonferroni correction). Cell attachment at 4 h for each membrane was analysed using paired *t*-test. *p*, 0.05 was accepted as the level of statistical significance.

## Figures and Tables

**Table 1:** Mechanical properties of the hydrogel membranes Chi_20_FG_20_ChS_5_Gly_55_, Chi_17_FG_34_ChS_4_Gly_45_, Chi_13_FG_50_ChS_3_Gly_34_ and Chi_13_FG_53_Gly_34_.

Specimen	Stress (MPa)	Strain (%)	% Change in Stress	% Change in Strain
Chi_20_FG_20_ChS_5_Gly_55_	2.64 ± 0.23	52.87 ± 2.61	−56.22	−65.43
Chi_17_FG_34_ChS_4_Gly_45_	3.33 ± 1.00	62.35 ± 1.46	−44.78	−59.24
Chi_13_FG_50_ChS_3_Gly_34_	2.89 ± 0.24	110.3 ± 7.75	−52.07	−27.88
Chi_13_FG_53_Gly_34_	6.03 ± 0.46	152.95 ± 8.85	reference	reference

**Table 2:** Code names and compositions of the hydrogel membranes.

Sample Code	Chitosan (*w*/*v*)	Fish Gelatin (*w*/*v*)	Chondroitin Sulfate (*w*/*v*)	Glycerol (*v*/*v*)
Chi_20_FG_20_ChS_5_Gly_55_ (% wt. 20/20/5/55)	2%	2%	0.03%	4%
Chi_17_FG_34_ChS_4_Gly_45_ (% wt. 17/34/4/45)	2%	4%	0.03%	4%
Chi_13_FG_50_ChS_3_Gly_34_ (% wt. 13/50/3/34)	2%	8%	0.03%	4%
Chi_30_Gly_70_ (% wt. 30/70)	2%	-	-	4%
Chi_13_FG_53_Gly_34_ (% wt. 13/53/34)	2%	8%	-	4%

## Data Availability

The datasets generated for this study are available on request to the corresponding author.
